# Uncoupling Systemic Inflammation from Body Mass Index: The Unseen Role of Visceral Adiposity and Metabolic Phenotypes—A Subgroup Analysis of the Nationwide OBREDI-TR Cohort

**DOI:** 10.3390/jcm15103794

**Published:** 2026-05-14

**Authors:** Kubilay İşsever, Alihan Oral, Ahmed Cihad Genç, Ihsan Solmaz, Nizameddin Koca, Ulas Serkan Topaloglu, Ismail Demir, Ahmet Dundar, Ali Kirik, Ozge Kama Basci, Hacer Sen, Emine Binnetoglu, Nalan Okuroglu, Ahmet Aydin, Zeynep Irmak Kaya, Hamit Yildiz, Aycan Acet, Gokhan Tazegul, Hasan Sozel, Osman Ozudogru, Selcuk Yaylacı, Ugur Bayram Korkmaz, Nur Duzen Oflas, Celalettin Küçük, Kamil Konur, Teslime Ayaz, Aysun Isiklar, Esref Arac, Hilmi Erdem Sumbul, Huseyin Ali Ozturk, Ali Burak Govez, Yusuf Usame Durmus, Atilla Onmez, Sibel Ocak Serin, Nazif Yalcin, Aysegul Ertinmaz, Alper Tuna Guven, Mehmet Kok, Yasin Sahinturk, Seyit Uyar

**Affiliations:** 1 Department of Internal Medicine, Faculty of Medicine, Giresun University, 28100 Giresun, Türkiye; kubilayissever@gmail.com; 2 Department of Internal Medicine, Faculty of Medicine, Biruni University, Halkalı Street No. 99, 34295 Istanbul, Türkiye; dr.alihanoral@gmail.com; 3 Department of Internal Medicine, Ahmed Cihad Genc Private Clinic, 34480 Istanbul, Türkiye; genccihad@gmail.com; 4 Department of Internal Medicine, Diyarbakir Gazi Yasargil Education Research Hospital, 21070 Diyarbakir, Türkiye; ihsan2157@gmail.com; 5 Department of Internal Medicine, Health Sciences University Bursa Health Application and Research Center, Bursa City Hospital, 16250 Bursa, Türkiye; nkoca@yahoo.com (N.K.); nazifyalcin16@gmail.com (N.Y.); aertinmaz@yahoo.com (A.E.); 6 Department of Internal Medicine, Kayseri City Hospital, 38080 Kayseri, Türkiye; ustop38@gmail.com; 7 Department of Internal Medicine, Bozyaka Education Research Hospital, 35170 Izmir, Türkiye; drismaildemir22@gmail.com; 8 Department of Internal Medicine, Mardin Savur Prof. Dr. Aziz Sancar State Hospital, 47860 Savur, Türkiye; drahmetdundar@hotmail.com; 9 Department of Internal Medicine, Faculty of Medicine, Balikesir University, Altieylül, 10145 Balikesir, Türkiye; alikirik87@hotmail.com (A.K.); ozgee.kama@gmail.com (O.K.B.); hcrgrsy@hotmail.com (H.S.); 10 Department of Internal Medicine, Corlu Vatan Hospital, 59860 Corlu, Türkiye; edemirbas1@yahoo.com; 11 Department of Internal Medicine, Fatih Sultan Mehmet Education Research Hospital, 34752 Istanbul, Türkiye; nokuroglu@yahoo.com; 12 Department of Internal Medicine, Faculty of Medicine, Medipol University, Bagcilar, 34214 Istanbul, Türkiye; uzm.dr.ahmetaydin@gmail.com; 13 Department of Internal Medicine, Health Sciences University Eskisehir Health Application and Research Center, Eskisehir City Hospital, 26080 Eskisehir, Türkiye; dr.zeynepirmak@gmail.com; 14 Department of Internal Medicine, Faculty of Medicine, Gaziantep University, 27600 Sehitkamil, Türkiye; drhyildiz@hotmail.com; 15 Department of Internal Medicine, Faculty of Medicine, Kutahya Health Sciences University, 43020 Kutahya, Türkiye; aycanacet80@icloud.com; 16 Department of Internal Medicine, Faculty of Medicine, Marmara University, 34854 Istanbul, Türkiye; drgtazegul@gmail.com (G.T.); alper.tuna.guven@gmail.com (A.T.G.); 17 Department of Internal Medicine, Faculty of Medicine, Akdeniz University, 07100 Antalya, Türkiye; hasansozel@akdeniz.edu.tr; 18 Department of Internal Medicine, Faculty of Medicine, Erzincan Binali Yildirim University, 24100 Erzincan, Türkiye; osmanozudogru2@gmail.com; 19 Department of Internal Medicine, Faculty of Medicine, Sakarya University, 54100 Sakarya, Türkiye; selcukyaylaci@sakarya.edu.tr; 20 Department of Internal Medicine, Izmir Katip Celebi Education Research Hospital, 35360 Izmir, Türkiye; ugurbk07@gmail.com; 21 Department of Internal Medicine, Faculty of Medicine, Van Yuzuncu Yil University, 54100 Van, Türkiye; dr.nurdzn@hotmail.com; 22 Vocational School, Biruni University, 34295 Istanbul, Türkiye; celalettinkucuk@yahoo.com; 23 Department of Internal Medicine, Faculty of Medicine, Recep Tayyip Erdogan University, 53020 Rize, Türkiye; 24 Department of Internal Medicine, Bakircay University Cigli Education Research Hospital, 36610 Izmir, Türkiye; drteslimeayaz@gmail.com; 25 Department of Internal Medicine, Acibadem Atasehir Hospital, Atasehir, 34642 Istanbul, Türkiye; aysunisiklar@gmail.com; 26 Department of Internal Medicine, Faculty of Medicine, Dicle University, 21010 Diyarbakir, Türkiye; esrefarac@gmail.com; 27 Department of Internal Medicine, Health Sciences University Adana Health Application and Research Center, Adana City Hospital, 01230 Adana, Türkiye; erdemsumbul@gmail.com (H.E.S.); drozturkhuseyinali@gmail.com (H.A.O.); abgovez@gmail.com (A.B.G.); drmusyusuf80@gmail.com (Y.U.D.); 28 Department of Internal Medicine, Faculty of Medicine, Duzce University, 81000 Duzce, Türkiye; attilaonmez@gmail.com; 29 Department of Internal Medicine, Umraniye Education Research Hospital, Umraniye, 34764 Istanbul, Türkiye; drsibelocak@gmail.com; 30 Department of Internal Medicine, Antalya Education Research Hospital, 07080 Antalya, Türkiye; dr.mehmetkok@hotmail.com (M.K.); drsahinturk@yahoo.com (Y.S.); seyituyar79@hotmail.com (S.U.)

**Keywords:** obesity, inflammatory indices, body mass index, visceral adiposity, systemic inflammation, OBREDI-TR

## Abstract

**Objectives:** Although obesity is known to cause low-grade chronic inflammation, the extent to which body mass index (BMI) reflects this remains questionable. To investigate this, we classified a national obesity cohort by BMI and evaluated its association with complete blood count (CBC)-derived systemic inflammatory indices. **Methods:** This retrospective, multi-center study included 6499 adults from the OBREDI-TR cohort with available laboratory data. Patients were categorized by BMI into Class I (30.0–34.9 kg/m^2^, *n* = 2751), Class II (35.0–39.9 kg/m^2^, *n* = 1804), and Class III (≥40.0 kg/m^2^, *n* = 1944) obesity. We compared demographic, clinical, and laboratory parameters, especially in terms of CBC-derived inflammation parameters: neutrophil-to-lymphocyte ratio (NLR), platelet-to-lymphocyte ratio (PLR), lymphocyte-to-monocyte ratio (LMR), systemic immune-inflammation index (SII), and systemic inflammation response index (SIRI). **Results:** The Class III group was younger (42.73 ± 13.21 vs. 45.14 ± 14.12 in Class I) and predominantly female (*p* < 0.001 for both). None of the evaluated inflammatory indices showed significant differences across the groups (NLR, *p* = 0.435; PLR, *p* = 0.141; LMR, *p* = 0.520; SII, *p* = 0.326; SIRI, *p* = 0.459). Interestingly, hypertension was less common in the Class III obesity group (49.0% vs. 53.5% in Class I, *p* = 0.009). **Conclusions:** The failure of increasing inflammatory indices to parallel BMI, creating a “ceiling effect,” reflects the inadequacy of BMI in determining inflammatory burden. Evaluating the inflammatory burden of obesity through visceral adiposity and metabolic phenotyping (metabolically healthy (MHO) vs. metabolically unhealthy obesity (MUO) rather than BMI will provide a more accurate basis for objective clinical evaluation and personalized treatment.

## 1. Introduction

Although many people worldwide became familiar with the word “pandemic” through the COVID-19 outbreak, it is an undeniable fact that the true “silent pandemic” of the past 50 years is type 2 diabetes and obesity [1]. According to current World Health Organization data, the presence of approximately 3 billion overweight and 1 billion obese individuals globally—with this prevalence doubling in just the last 30 years—has turned this silent pandemic into an urgent public health crisis [2]. For many years, parameters such as body mass index (BMI ≥ 30 kg/m^2^) and waist circumference (using population-specific thresholds) have been used to diagnose obesity. However, recent studies argue that these conventional parameters can be affected by various conditions and often fail to capture underlying adipose tissue dysfunction. Instead, phenotypic definitions—such as visceral adiposity, ectopic fat accumulation, and metabolically healthy or metabolically unhealthy obesity (MHO, MUO)—better reflect the true complication burden of obesity [3,4]. Obesity has been shown to cause insulin resistance, type 2 diabetes, hypertension, dyslipidemia, and microalbuminuria (components of metabolic syndrome), while also increasing the risk of numerous complications such as cancer, obstructive sleep apnea syndrome (OSAS), osteoarthritis, fatty liver disease, coronary artery disease, and congestive heart failure [5,6]. Considering all these complications, controlling and managing this pandemic—which places a massive burden on healthcare expenditures alongside increased mortality and morbidity—requires it to be very well defined in the first place [7].

In individuals defined as “obese,” increased adipose tissue leads over time to altered secretion of hormones such as leptin and adiponectin, alongside the release of pro-inflammatory cytokines like tumor necrosis factor-alpha (TNF-α) and interleukin-6 (IL-6), and reactive oxygen species [8]. The chronic low-grade inflammation in obesity, initiated by these steps, eventually drives the process that leads to the aforementioned complications through lipotoxicity, insulin and leptin resistance, fibrogenesis, and pancreatic beta-cell dysfunction [9]. To clinically detect this inflammation, in addition to classic acute-phase reactants such as plasma C-reactive protein (CRP), high-sensitivity CRP (hs-CRP), ferritin, erythrocyte sedimentation rate, and albumin, systemic inflammatory parameters derived from complete blood counts can also be utilized [10,11,12]. Standing out with the advantages of being simple, rapidly calculable, and analyzable within minutes from a single tube of blood available in most healthcare facilities, these parameters have been found to guide diagnosis and prognosis in numerous diseases to date. The literature also includes studies suggesting their potential utility in the diagnosis, classification, and prognosis of obesity [10,11]. For instance, recent evidence demonstrates that complete blood count-derived indices, particularly the systemic immune-inflammation index (SII) and neutrophil-to-lymphocyte ratio (NLR), are significantly correlated with regional body fat distribution, visceral adiposity, and the severity of metabolic syndrome, confirming their value as accessible biomarkers for assessing the inflammatory burden of obesity [13].

Discoveries in the pathophysiology of obesity and visceral adiposity have led to discussions regarding classical definitions and classifications [14]. As studies increasingly demonstrate that some individuals with a normal body mass index carry a high complication risk due to increased visceral adiposity, whereas some individuals with a high BMI face a lower complication risk due to reduced visceral adiposity—reflecting the concepts of metabolically healthy (MHO) and metabolically unhealthy obesity (MUO)—the descriptive and predictive value of BMI has begun to be questioned [15]. Therefore, in this study, we aimed to determine whether the classification of obesity by BMI is associated with complete blood count-derived systemic inflammatory indices in a large national patient cohort [16].

## 2. Materials and Methods

### 2.1. Study Design and Population

This study is a subgroup analysis of the original OBREDI-TR cohort—a retrospective, cross-sectional study, comprising patients from the initial 10,121-person cohort—who had available laboratory data at the time of admission [16]. The national, multicenter OBREDI-TR study, which was conducted between December 2023 and December 2024, originally included patients aged 18 years and older with a BMI ≥ 30 kg/m^2^, while excluding those with a history of any malignancy, type 1 diabetes mellitus, or secondary obesity of genetic or endocrine origin. Furthermore, to prevent potential confounding in our analysis, we additionally excluded individuals with conditions known to alter plasma levels of inflammatory markers, such as acute or chronic active infections, and active acute or chronic rheumatologic or autoimmune diseases. Following these rigorous exclusion criteria, a total of 6499 patients (4681 females and 1818 males) were enrolled in the present subgroup analysis. While the original study protocol was approved by the Ethics Committee of Biruni University (Decision No: 2024/84, Date: 19 November 2024), additional ethical approval for this specific analysis was obtained from the Ethics Committee of Giresun University (Decision No: 23.07.2025/21, Date: 23 July 2025). This study was conducted in accordance with the ethical principles of the Helsinki Declaration.

### 2.2. Data Collection

The demographic data, anthropometric and vital sign measurements of the patients, and the diagnostic criteria used for their comorbidities were detailed in the original OBREDI-TR study (16). Patients were categorized by BMI into Class I (30.0–34.9 kg/m^2^, *n* = 2751), Class II (35.0–39.9 kg/m^2^, *n* = 1804), and Class III (40.0 kg/m^2^, *n* = 1944) obesity. In addition, the analysis of blood tests obtained from the patients at their initial outpatient visit was included in the dataset for this subgroup analysis. To ensure that the CBC-derived indices accurately reflected the chronic low-grade inflammatory burden of obesity rather than short-term physiological fluctuations, strict standardization principles were applied to the timing and clinical conditions of blood sampling. All patients were evaluated during a clinically stable period in the outpatient setting; individuals presenting with acute symptoms or severe physiological stress or those requiring immediate treatment adjustments were inherently excluded from this routine screening cohort. Furthermore, all venous blood samples were collected in the morning following an 8- to 12-h overnight fast, simultaneously with the assessment of fasting plasma glucose and lipid profiles. The included laboratory data comprised complete blood count (CBC) parameters, as well as fasting plasma glucose, urea, creatinine, uric acid, alanine aminotransferase (ALT), aspartate aminotransferase (AST), and a lipid panel including low-density lipoprotein cholesterol (LDL-C), high-density lipoprotein cholesterol (HDL-C), triglycerides, and total cholesterol.

Systemic inflammatory indices were calculated based on the absolute cell counts (expressed as 10^3^/L) obtained from the CBC, according to the following established formulas:

Neutrophil-to-Lymphocyte Ratio (NLR): Calculated by dividing the absolute neutrophil count by the absolute lymphocyte count (NLR = N/L) [17].

Platelet-to-Lymphocyte Ratio (PLR): Calculated by dividing the absolute platelet count by the absolute lymphocyte count (PLR = P/L) [17].

Lymphocyte-to-Monocyte Ratio (LMR): Calculated by dividing the absolute lymphocyte count by the absolute monocyte count (LMR = L/M) [17].

Systemic Immune-Inflammation Index (SII): Calculated by multiplying the absolute platelet count by the absolute neutrophil count, divided by the absolute lymphocyte count (SII = P × N/L) [17].

Systemic Inflammation Response Index (SIRI): Calculated by multiplying the absolute neutrophil count by the absolute monocyte count, divided by the absolute lymphocyte count (SIRI = N × M/L) [18].

### 2.3. Statistical Analysis

The normality of continuous variables across the three independent groups was evaluated using the Kolmogorov–Smirnov and Shapiro–Wilk tests, supplemented by visual inspections of histograms and probability plots. Continuous variables were expressed as mean ± standard deviation, while categorical variables were presented as numbers (*n*) and percentages (%). For comparisons among the three independent groups, one-way analysis of variance (ANOVA) was utilized for continuous variables that met the normal distribution assumption. When ANOVA indicated a statistically significant difference, post-hoc analyses were conducted to determine the specific intergroup differences. Categorical variables were compared using the Chi-square test. If statistically significant differences were detected in the primary outcomes, multivariate analyses were planned to identify potential confounding factors and assess independent effects. Multinomial logistic regression analysis was performed to identify independent predictors of obesity classes. Variables included in the model were selected based on clinical relevance, potential confounding effects, and findings from previous literature, rather than solely on univariate statistical significance. Adjusted odd ratios (ORs) with 95% confidence intervals (CIs) were calculated. Statistical significance was set at *p* < 0.05. All statistical analyses were performed using the IBM SPSS Statistics version 26.0 software package (IBM Corp., Armonk, NY, USA).

## 3. Results

The demographic, anthropometric, and laboratory characteristics of the study population are summarized in Table 1. Of the 6499 patients included, approximately 42.32% were categorized as having Class I obesity, 27.75% as Class II, and 29.91% as Class III. Females accounted for 72.03% of the overall cohort, and this female predominance significantly intensified with advancing obesity classes (65.39%, 72.95%, and 80.56% from Class I to III, respectively; *p* < 0.001). As anticipated, body weight, BMI, and waist circumference increased significantly with higher obesity classes, while height significantly decreased (*p* < 0.001 for all). Mean diastolic blood pressure also showed a correlated increase with the obesity class (*p* < 0.001). Interestingly, the highest mean systolic blood pressure was observed in Class II obesity, whereas the lowest was recorded in Class III (*p* < 0.001). Among the laboratory parameters, no significant differences were observed across the groups regarding fasting blood glucose, hemoglobin A1c, creatinine, liver function tests, thyroid-stimulating hormone, uric acid, isolated complete blood count parameters, or any of the five indices indicating systemic inflammation. Within the lipid panel, only high-density lipoprotein cholesterol (HDL-C) levels differed significantly, being highest in Class III and lowest in Class II obesity (*p* = 0.042). Waist-to-height ratios (WHtRs) were also calculated for all patients. A cut-off value of 0.6 was utilized, as the 2025 National Institute for Health and Care Excellence (NICE) guidelines classify values at or above this threshold as indicating “high central adiposity” [19]. Overall, 81.3% of the cohort met or exceeded this threshold. Furthermore, the prevalence of high central adiposity increased significantly with advancing obesity classes (66.6%, 87.6%, and 96.2% for Class I, II, and III, respectively; *p* < 0.001).

The clinical characteristics and comorbidities of the patients according to BMI classes are presented in Table 2. In this analysis evaluating various parameters—including smoking status, type 2 diabetes mellitus, hypertension, dyslipidemia, coronary heart disease, obstructive sleep apnea syndrome, and hepatosteatosis—only the prevalence of hypertension was found to significantly decrease in an inverse manner as the obesity class advanced (*p* = 0.009). No statistically significant differences were observed among the groups regarding the other clinical parameters and comorbidities.

To account for potential confounding factors, a multinomial logistic regression analysis was conducted, with intergroup comparisons structured as ‘Class II vs. Class I’ and ‘Class III vs. Class I’ (Table 3). The results demonstrated that parameters such as age, sex, waist circumference, hypertension prevalence, and systolic and diastolic blood pressure—which initially exhibited significant differences among the groups—remained significantly different. Interestingly, the previously observed difference in HDL cholesterol levels lost its statistical significance in this adjusted model. Furthermore, the systemic inflammatory indices along with smoking status, which initially showed no significant differences across the groups, maintained their non-significant status following this analysis.

## 4. Discussion

In our study, inflammatory indices such as SII, SIRI, and NLR—well-known for their strong association with metabolic syndrome and insulin resistance—did not differ significantly across obesity classes (Class I, II, and III). This finding contrasts with existing literature that compares obese cohorts to healthy, normal-weight controls, where a linear relationship between inflammatory indices and metabolic deterioration is typically reported. For instance, a recent study by Nicoară et al. demonstrated that SII and SIRI were significantly elevated in obese children with metabolic syndrome, highlighting their high diagnostic value [20]. However, when directly comparing established obesity classes, our findings indicate that the systemic inflammatory burden does not increase indefinitely with BMI. This phenomenon can be explained by a ‘ceiling effect’ hypothesis inherent to obesity pathophysiology [21]. As demonstrated by Tremblay et al., adipocyte hypertrophy—the primary driver of adipose tissue inflammation—increases linearly with overall adiposity but reaches a plateau once BMI exceeds the 30 kg/m^2^ threshold [21]. In the setting of Class I obesity (BMI ≥ 30 kg/m^2^), tissue-level hypoxia and macrophage infiltration likely reach their functional limits, causing the systemic inflammatory response to become saturated. Furthermore, the true determinant of systemic inflammation is visceral adiposity rather than BMI, as the latter is a surrogate metric susceptible to confounding factors such as fluid retention [21,22]. Consequently, our results suggest that BMI-based classification alone fails to accurately reflect the systemic inflammatory burden, implying that the inflammatory threshold in obese patients is likely already met at the Class I obesity stage.

From a clinical standpoint, the lack of significant variance in these indices across advancing BMI classes raises an important question regarding their routine utility. Our findings suggest a dual inadequacy: not only is BMI a poor surrogate for reflecting the true inflammatory burden of obesity, but CBC-derived inflammatory indices themselves may also have limited capacity as standalone markers for discriminating the severity of inflammation in advanced obesity classes. While indices like NLR, PLR, SII, and SIRI are highly practical, accessible, and well-correlated with initial metabolic deterioration, they are relatively indirect and crude markers of systemic inflammation once the adiposity threshold is breached. Therefore, in daily clinical practice, a two-fold paradigm shift is necessary. First, clinicians should avoid evaluating the severity of obesity solely through the lens of BMI and instead prioritize objective measures of visceral adiposity. Second, CBC-derived indices should not be relied upon as absolute, solitary determinants of systemic inflammatory severity in patients with obesity. To obtain a more comprehensive and accurate cardiometabolic risk profile, these practical indices should be evaluated in conjunction with classical, robust pro-inflammatory biomarkers such as high-sensitivity C-reactive protein (hs-CRP) and interleukin-6 (IL-6) [8,12].

Another interesting finding in our cohort was the paradoxical increase in HDL cholesterol and the decline in hypertension prevalence as obesity class advanced. Our “demographic heterogeneity” hypothesis might explain this shift and clarify not only the unexpected results in hypertension and HDL but also the plateau observed in inflammatory indices. Specifically, the Class III obesity group was relatively younger and showed a distinct female predominance (over 80%). Given that the age difference between Class III and Class I was marginal—only about two years (42.73 ± 13.1 vs. 45.14 ± 14.12 years, respectively)—the primary driver of this metabolic variation appears to be the high proportion of female patients. This demographic trend is well-supported by large-scale epidemiological data from bariatric surgery cohorts. Similarly to our study, Tremblay et al. reported that women comprised nearly 70% of a severe obesity cohort and sought treatment at a significantly younger age than men [21]. When evaluating metabolic outcomes between sexes, this female-dominant profile heavily influences systemic risk. The same study showed that men with severe obesity had a profoundly worse lipid profile and nearly double the incidence of hypertension (48.8% vs. 26.4%) compared to women, while women maintained significantly higher HDL levels [21]. In our cohort, the initial significance of the HDL differences between the groups disappeared after adjusting for age and sex in our multivariable logistic regression analysis. Thus, a more cautious approach should be implemented while interpreting the HDL trends across obesity classes, particularly given the lack of data regarding lipid-lowering therapies in our study.

The underlying pathophysiology behind the unexpected results of our study might involve sexual dimorphism in adipose tissue distribution. Men tend to store fat in pro-inflammatory visceral and perivascular (perivascular adipose tissue—PVAT) depots, which directly trigger hypertension and vascular stiffness. In contrast, the synergistic effect of younger age and female sex hormones favors fat deposition in cardio-protective subcutaneous and gluteofemoral regions [22,23,24]. As a result, the shift toward a female-dominated demographic in our Class III cohort may mask the metabolic deterioration normally expected with weight gain. This masking could potentially explain why advancing obesity class paradoxically resulted in a ceiling effect for inflammatory indices, alongside improved HDL and reduced hypertension. Therefore, relying solely on BMI for defining and managing obesity might be inadequate, as it may fail to account for this metabolic masking. Clinical practice would benefit from using parameters that objectively assess visceral adiposity. Modalities that accurately quantify regional fat distribution, such as dual-energy X-ray absorptiometry (DXA) or bioelectrical impedance analysis, align much better with the severity of low-grade chronic inflammation and actual cardiometabolic risk [23,25].

A parallel and equally intriguing hemodynamic paradox in our cohort was the divergent trajectory of blood pressure components across obesity classes: increasing BMI correlated with a significant rise in mean diastolic blood pressure alongside a drop in systolic pressure, ultimately narrowing the pulse pressure in Class III obesity. This physiological shift likely stems from the interplay between our cohort’s demographic distribution and obesity-induced vascular remodeling. Typically, the widening of pulse pressure—characterized by surging systolic and declining diastolic values—is a hallmark of central arterial stiffening driven by advancing age and prolonged metabolic insult [22]. In line with this, the Class I and II groups in our cohort, which exhibited higher systolic and pulse pressures, were significantly older. Conversely, the younger, predominantly female makeup of the Class III group suggests a relative preservation of large-artery compliance, effectively blunting any systolic surge. However, the concurrent—albeit minimal—yet significant elevation in diastolic pressure within this severe obesity tier likely reflects a state of heightened peripheral vascular resistance. As outlined by Koenen et al., the expansion of PVAT during severe obesity strips the tissue of its inherent vasodilatory properties. This dysfunctional PVAT shifts toward a pro-inflammatory phenotype, thereby increasing local microvascular tone and peripheral resistance—hemodynamic changes that predominantly drive up diastolic blood pressure [22]. Ultimately, the narrowed pulse pressure observed in our Class III cohort appears to capture a highly specific pathophysiological window: an early phase of PVAT-induced peripheral resistance (marked by lower systolic and higher diastolic pressures) that precedes the onset of age-related macroscopic arterial stiffening.

A key methodological strength of our study is the homogeneous distribution of smoking status and major comorbidities—including type 2 diabetes mellitus (T2DM), dyslipidemia, and coronary heart disease—across the three obesity classes. The absence of significant intergroup differences in these parameters establishes a robust clinical baseline that effectively mitigates confounding bias. This clinical uniformity strengthens the reliability of our primary findings, ensuring that the plateau in systemic inflammatory indices and the divergent hemodynamic shifts accurately reflect obesity-driven pathophysiology rather than an artifact of unequal comorbidity burdens. Notably, the prevalence of major metabolic comorbidities like T2DM and dyslipidemia did not scale proportionally with escalating BMI. This flat trajectory underlines a pivotal concept in modern metabolic research: the fundamental limitation of relying on BMI as an isolated prognostic marker [23,26]. Current literature confirms that cardiometabolic risk in chronic obesity is dictated primarily by visceral adipose tissue (VAT) dysfunction, ectopic fat deposition, and adipocyte hypertrophy, rather than sheer total body weight [21,27]. Our data strongly support this paradigm, implying that once the initial obesity threshold is breached, systemic comorbidity rates depend heavily on qualitative adipose dysfunction and regional fat distribution instead of continuous linear BMI expansion.

Beyond adipose tissue dynamics, an emerging and critical dimension in this context is the role of the gut microbiota in modulating systemic inflammation. Increasing evidence suggests that gut dysbiosis significantly contributes to low-grade chronic inflammation and metabolic dysfunction, largely independent of BMI. Consequently, microbiota-targeted therapeutic strategies, such as fecal microbiota transplantation (FMT), may offer novel translational avenues for mitigating the inflammatory burden in severe obesity [28]. However, further randomized controlled trials are required to definitively establish the clinical efficacy and the exact role of these novel approaches in the management of obesity.

Our study has several notable strengths and inherent limitations. The inclusion of a large, multicenter cohort comprising 6499 patients and the homogeneous distribution of major comorbidities across groups constitute the primary methodological strengths of our analysis. Furthermore, utilizing cost-effective, non-invasive, and universally accessible CBC-derived inflammatory indices (such as SII, SIRI, and NLR) within such an extensive severe obesity cohort significantly enhances the everyday clinical applicability of our findings. To the best of our knowledge, this is one of the most comprehensive real-world studies to date evaluating the ‘ceiling effect’ of systemic inflammation across advancing classes of severe obesity. By leveraging a massive national dataset and challenging the standalone prognostic value of BMI, our findings provide a robust epidemiological foundation that strongly encourages a clinical paradigm shift toward phenotype-based obesity management. Nevertheless, certain limitations must be acknowledged. The retrospective and cross-sectional design precludes the establishment of definitive causal relationships between advancing obesity and the observed metabolic shifts. Furthermore, obesity is a chronic and dynamic metabolic condition that evolves over decades. Therefore, attempting to characterize its long-standing inflammatory burden using a single laboratory snapshot—such as one-time CBC-derived indices—carries intrinsic conceptual limitations. It is highly plausible that our negative findings reflect not only the inadequacy of BMI as a metabolic classifier, but also the inherent limitations of using point-in-time markers to represent such a biologically complex disease trajectory. Additionally, while our cohort is undeniably large and multicenter, it is a retrospective dataset assembled from various institutions rather than a prospectively structured national registry. Consequently, this design inherently introduces potential limitations regarding the strict standardization, uniformity of laboratory measurements, and consistency of data capture across the different participating sites. The study population is restricted to a single nationality, which may limit the generalizability of the results to other ethnic groups. Methodologically, relying on electronic health records and lacking a non-obese control arm (BMI < 30 kg/m^2^) restrict broader physiological comparisons, making it difficult to definitively contextualize whether our findings represent a true inflammatory “ceiling effect” or simply a homogeneous inflammatory baseline across all obese classes. We were also unable to objectively quantify visceral adiposity using advanced modalities like bioelectrical impedance analysis or dual-energy X-ray absorptiometry (DXA). Another critical limitation is the unavailability of detailed pharmacological data—specifically the use of antihypertensive and lipid-lowering medications—and specific tests to measure arterial stiffness such as pulse wave velocity, which introduces potential confounding bias regarding the trajectories of lipid profiles and blood pressure values. Finally, while we mechanistically attribute our clinical findings to phenomena such as the inflammatory ‘ceiling effect’ and PVAT dysfunction, we lack direct serological or histological validation; specific adipokines (e.g., adiponectin, leptin) and pro-inflammatory cytokines (e.g., TNF-α, IL-6) were not measured in our cohort.

## 5. Conclusions

Ultimately, our findings establish that body mass index fails to adequately capture the low-grade chronic inflammatory burden inherent to obesity. For accurate diagnosis, risk stratification, and clinical management, routine practice must shift away from relying solely on BMI toward alternative metrics that directly quantify visceral adiposity. Validating these observations and integrating them into future clinical guidelines will require large-scale prospective cohorts and randomized controlled trials.

## Figures and Tables

**Table 1:** Demographic, anthropometric, and laboratory characteristics of the study population by BMI classes.

Variables	Class I Obesity (BMI 30.0–34.9 kg/m^2^, *n* = 2751)	Class II Obesity (BMI 35.0–39.9 kg/m^2^, *n* = 1804)	Class III Obesity (BMI ≥ 40.0 kg/m^2^, *n* = 1944)	Total Cohort (*n* = 6499)	*p*-Value
**Age (years)**	45.14 ± 14.12	44.03 ± 13.68	42.73 ± 13.21	44.11 ± 13.77	<0.001 ^a^
**Gender, *n* (%)**					<0.001 ^a^
Female	1799 (65.39%)	1316 (72.95%)	1566 (80.56%)	4681 (72.03%)	
Male	952 (34.61%)	488 (27.05%)	378 (19.44%)	1818 (27.97%)	
**Weight (kg)**	88.45 ± 11.10	100.67 ± 12.19	117.32 ± 17.31	100.48 ± 18.14	<0.001 ^a^
**Height (cm)**	165.51 ± 9.48	164.17 ± 9.22	161.67 ± 9.04	163.99 ± 9.42	<0.001 ^a^
**BMI (kg/m^2^)**	32.06 ± 1.50	37.21 ± 1.42	44.91 ± 4.63	37.33 ± 6.07	<0.001 ^a^
**Waist circumference (cm)**	104.52 ± 10.80	111.97 ± 11.10	122.42 ± 13.86	111.94 ± 14.04	<0.001 ^a^
**Systolic BP (mmHg)**	136.42 ± 22.72	139.54 ± 24.72	127.35 ± 39.54	134.57 ± 29.66	<0.001 ^a^
**Diastolic BP (mmHg)**	80.08 ± 7.88	81.19 ± 8.16	81.77 ± 8.62	80.89 ± 8.22	<0.001 ^b^
**Fasting glucose (mg/dL)**	111.22 ± 41.91	112.18 ± 44.35	111.73 ± 42.63	111.64 ± 42.81	0.762
**HbA1c (%)**	6.24 ± 1.37	6.28 ± 1.43	6.28 ± 1.47	6.26 ± 1.42	0.687
**Platelets (10^3^/µL)**	285.98 ± 72.59	283.99 ± 75.06	282.46 ± 73.00	284.38 ± 73.41	0.261
**Hemoglobin (g/dL)**	13.52 ± 1.84	13.46 ± 1.72	13.49 ± 1.74	13.49 ± 1.78	0.606
**Lymphocytes (10^3^/µL)**	2.51 ± 0.81	2.53 ± 0.83	2.53 ± 0.87	2.52 ± 0.83	0.625
**Monocytes (10^3^/µL)**	0.56 ± 0.25	0.55 ± 0.24	0.55 ± 0.23	0.55 ± 0.24	0.251
**Neutrophils (10^3^/µL)**	4.82 ± 2.20	4.81 ± 1.97	4.73 ± 1.92	4.79 ± 2.06	0.275
**Urea (mg/dL)**	25.27 ± 14.19	25.19 ± 13.86	25.38 ± 13.85	25.28 ± 14.00	0.923
**Creatinine (mg/dL)**	0.80 ± 0.35	0.79 ± 0.35	0.79 ± 0.29	0.79 ± 0.33	0.426
**ALT (U/L)**	27.72 ± 22.89	26.16 ± 20.56	26.95 ± 23.95	27.06 ± 22.60	0.075
**AST (U/L)**	23.35 ± 16.78	22.91 ± 16.58	23.25 ± 15.80	23.20 ± 16.44	0.670
**Triglycerides (mg/dL)**	161.66 ± 96.67	162.58 ± 111.29	162.40 ± 93.82	162.14 ± 100.15	0.948
**LDL-C (mg/dL)**	121.46 ± 37.00	121.04 ± 37.17	121.25 ± 35.60	121.28 ± 36.63	0.932
**HDL-C (mg/dL)**	48.56 ± 14.29	47.85 ± 12.82	49.09 ± 16.81	48.52 ± 14.71	**0.042**
**Total cholesterol (mg/dL)**	201.27 ± 93.26	197.33 ± 45.02	202.76 ± 101.89	200.62 ± 85.70	0.146
**Uric acid (mg/dL)**	5.24 ± 2.19	5.24 ± 3.12	5.42 ± 3.93	5.30 ± 3.06	0.160
**TSH (µIU/mL)**	2.68 ± 9.96	3.53 ± 21.96	4.03 ± 50.49	3.32 ± 30.70	0.334
**NLR**	2.15 ± 1.81	2.08 ± 1.42	2.12 ± 2.46	2.12 ± 1.94	0.435
**PLR**	126.08 ± 73.15	122.27 ± 53.56	123.50 ± 67.66	124.25 ± 66.57	0.141
**LMR**	5.06 ± 3.01	5.15 ± 3.02	5.14 ± 2.56	5.11 ± 2.89	0.520
**SII**	611.26 ± 546.46	589.88 ± 506.88	590.65 ± 618.88	599.16 ± 558.84	0.326
**SIRI**	1.25 ± 1.75	1.16 ± 1.00	1.34 ± 7.57	1.25 ± 4.32	0.459

Data are presented as mean ± standard deviation for continuous variables and number (percentage) for categorical variables. BP: Blood pressure; BMI: Body mass index; LDL-C: Low-density lipoprotein cholesterol; HDL-C: High-density lipoprotein cholesterol; ALT: Alanine aminotransferase; AST: Aspartate aminotransferase; TSH: Thyroid-stimulating hormone; NLR: Neutrophil-to-lymphocyte ratio; PLR: Platelet-to-lymphocyte ratio; LMR: Lymphocyte-to-monocyte ratio; SII: Systemic immune-inflammation index; SIRI: Systemic inflammation response index. ^a^ Statistically significant differences observed across all three groups (*p* < 0.05 in post-hoc analysis). ^b^ Statistically significant difference observed between Class I vs. Class II and Class I vs. Class III (*p* < 0.05 in post-hoc analysis).

**Table 2:** Clinical characteristics and comorbidities of the study population by BMI classes.

Variables	Class I Obesity (BMI 30.0–34.9 kg/m^2^, *n* = 2751)	Class II Obesity (BMI 35.0–39.9 kg/m^2^, *n* = 1804)	Class III Obesity (BMI ≥ 40.0 kg/m^2^, *n* = 1944)	Total Cohort (*n* = 6499)	*p*-Value
**Smoking status, *n* (%)**					0.622
Never smoked	1491 (54.20%)	964 (53.44%)	1075 (55.30%)	3530 (54.32%)	
Former smoker	372 (13.52%)	237 (13.14%)	267 (13.73%)	876 (13.48%)	
Current smoker	888 (32.28%)	603 (33.43%)	602 (30.97%)	2093 (32.20%)	
**Type 2 diabetes mellitus, *n* (%)**	944 (34.31%)	625 (34.65%)	636 (32.72%)	2205 (33.93%)	0.392
**Hypertension, *n* (%)**	1472 (53.51%)	927 (51.39%)	952 (48.97%)	3351 (51.56%)	**0.009** **^a^**
**Dyslipidemia, *n* (%)**	2358 (85.71%)	1541 (85.42%)	1660 (85.43%)	5559 (85.55%)	0.949
**Hyperlipidemia, *n* (%)**	1834 (66.67%)	1164 (64.52%)	1264 (65.02%)	4262 (65.58%)	0.272
**Coronary heart disease, *n* (%)**	301 (10.94%)	227 (12.58%)	238 (12.24%)	766 (11.79%)	0.185
**Obstructive lung disease, *n* (%)**	298 (10.83%)	177 (9.81%)	207 (10.65%)	682 (10.49%)	0.528
**OSAS, *n* (%)**	161 (5.85%)	105 (5.82%)	98 (5.04%)	364 (5.60%)	0.439
**Abdominal ultrasound performed, *n* (%)**	1490 (54.16%)	979 (54.27%)	1061 (54.58%)	3530 (54.32%)	0.960
**Hepatosteatosis, *n* (%)**	1164 (73.39%)	734 (70.85%)	830 (73.58%)	2728 (72.75%)	0.271
**Gallstones, *n* (%)**	251 (15.97%)	140 (13.66%)	190 (16.93%)	581 (15.62%)	0.100

Data are presented as numbers (percentage). BMI: Body Mass Index; OSAS: Obstructive Sleep Apnea Syndrome. ^a^ Statistically significant difference observed predominantly between Class I vs. Class III (*p* < 0.05 in post-hoc analysis).

**Table 3:** Multinomial logistic regression analysis for independent predictors of obesity classes.

Variable	Class 2 vs. Class 1 OR (95% CI)	*p*-Value	Class 3 vs. Class 1 OR (95% CI)	*p*-Value
**Age**	0.990 (0.985–0.995)	**<0.001**	0.982 (0.976–0.989)	**<0.001**
**Male sex**	2.282 (1.969–2.644)	**<0.001**	5.644 (4.678–6.809)	**<0.001**
**Waist circumference (cm)**	1.069 (1.063–1.076)	**<0.001**	1.150 (1.141–1.158)	**<0.001**
**Systolic BP (mmHg)**	1.004 (0.999–1.008)	0.091	0.953 (0.948–0.958)	**<0.001**
**Diastolic BP (mmHg)**	1.012 (1.004–1.021)	**0.003**	1.040 (1.029–1.050)	**<0.001**
**HDL (mg/dL)**	0.996 (0.991–1.001)	0.095	1.000 (0.995–1.005)	0.982
**Hypertension**	1.045 (0.904–1.209)	0.551	0.627 (0.525–0.748)	**<0.001**
**NLR**	1.007 (0.902–1.123)	0.904	0.995 (0.885–1.117)	0.927
**LMR**	1.008 (0.981–1.036)	0.544	1.011 (0.982–1.041)	0.452
**PLR**	0.999 (0.997–1.000)	0.132	0.999 (0.998–1.001)	0.532
**SII**	1.000 (1.000–1.000)	0.510	1.000 (0.999–1.000)	0.634
**SIRI**	0.952 (0.878–1.032)	0.229	1.013 (0.987–1.040)	0.321
**Smoking (Never vs. Ref)**	0.993 (0.860–1.146)	0.921	1.164 (0.979–1.383)	0.085
**Smoking (Former vs. Ref)**	0.917 (0.745–1.130)	0.418	1.064 (0.831–1.362)	0.621

Note: OR = Odds Ratio; CI = Confidence Interval. Reference category is Class 1 Obesity. BP: Blood pressure; HDL: High-density lipoprotein cholesterol; NLR: Neutrophil-to-lymphocyte ratio; LMR: Lymphocyte-to-monocyte ratio; PLR: Platelet-to-lymphocyte ratio; SII: Systemic immune-inflammation index; SIRI: Systemic inflammation response index. Reference category for smoking is Current Smoker.

## Data Availability

The datasets generated and analyzed during the current study are available from the corresponding author upon reasonable request.

## Abbreviations

The following abbreviations are used in this manuscript:

ALT	Alanine aminotransferase
AST	Aspartate aminotransferase
BMI	Body mass index
BP	Blood pressure
CBC	Complete blood count
CRP	C-reactive protein
HDL-C	High-density lipoprotein cholesterol
hs-CRP	High-sensitivity C-reactive protein
IL-6	Interleukin-6
LDL-C	Low-density lipoprotein cholesterol
LMR	Lymphocyte-to-monocyte ratio
MHO	Metabolically healthy obesity
MUO	Metabolically unhealthy obesity
NLR	Neutrophil-to-lymphocyte ratio
OBREDI-TR	Obesity Related Diseases in Türkiye
OSAS	Obstructive sleep apnea syndrome
PLR	Platelet-to-lymphocyte ratio
SII	Systemic immune-inflammation index
SIRI	Systemic inflammation response index
T2DM	Type 2 diabetes mellitus
TNF-α	Tumor necrosis factor-alpha
TSH	Thyroid-stimulating hormone
VAT	Visceral adipose tissue
